# Editorial: Distributed training and rural health professions education

**DOI:** 10.3389/fmed.2025.1704188

**Published:** 2025-10-14

**Authors:** Roger Peter Strasser, Julian Wright, Mohamed Elhassan Abdalla, Lachlan Van Schaik, Jana Vera Muller

**Affiliations:** ^1^Northern Ontario School of Medicine University, Sudbury, ON, Canada; ^2^University of Waikato, Hamilton, New Zealand; ^3^The University of Melbourne, Melbourne, VIC, Australia; ^4^University of Limerick School of Medicine, Limerick, Ireland; ^5^Stellenbosch University, Stellenbosch, South Africa

**Keywords:** distributed training, rural health, health professions education, social accountability, community engagement, rural career pathways, place based education, Longitudinal Integrated Clerkships (LICs)

## Introduction

Since the early 20th century, most medical and other health professions education around the world has been predominantly based in large acute hospitals, known as teaching hospitals or academic health centers (1). In the latter half of the 20th century, there were moves to focus health professions education outside hospitals into the community, initially through Community Oriented Medical Education and subsequently Community Based Medical Education (2). During the same timeframe, population trends and changes in health service delivery contributed to increasing inequities for people living in remote and rural communities, particularly in access to healthcare (3). By the early 21st century, social accountability and community engagement were recognized as transformative trends in health professions education (HPE), aiming to ensure equitable access to high-quality healthcare delivered by well-trained professionals (4, 5). The 2010 *Lancet* Commission on Education of Health Professionals for the 21st Century recommended that academic institutions build strong relations with communities to provide a context for education programs focused on achieving health equity (6).

Fifteen years later, HPE is still predominantly based in large city teaching hospitals (7) and many remote and rural communities experience challenges accessing healthcare with shortages of skilled health workforce a major contributing factor (8). There have however been efforts internationally to ensure HPE takes place across a wide distribution of healthcare sites, including rural areas (9). In this context, *Frontiers in Medicine* initiated a Research Topic on Distributed Training and Rural Health Professions Education. This Research Topic attracted 18 manuscripts that are published in this Research Topic drawn from around the world, including three from low- and middle-income countries (LMIC), three articles from Europe, five papers from North America and seven articles from Australia. These 18 publications consist of: nine Original Research articles; two Brief Research Reports; three Curriculum, Instruction and Pedagogy papers; two Scoping/Systematic Literature Reviews; two Perspectives; and one Community Case Study. Ten of the articles focus on undergraduate HPE with three articles on postgraduate training, three research papers, and two articles that focus on community capacity building. Major themes that emerged from the articles are set out in the following sections.

## Fostering rural community capability

In post-conflict Colombia, Lombo-Caicedo et al. report on the success of a community-based intervention aimed at strengthening the competencies of informal caregivers in remote rural communities. This model of distributed training was found to be empowering and community building, with caregivers gaining not only knowledge and skills, but also enhanced self-efficacy and status in the community.

Scotland has developed Community Training Hubs in the remote, rural and island context toward addressing recruitment and retention challenges in the primary care workforce, particularly general practitioners, advanced nurse practitioners, pharmacists and practice nurses. In their Brief Research Report, Munoz et al. present the development of their proposed evaluation framework designed to demonstrate the effectiveness of the Hub strategy in attracting and retaining talent, as well as reducing social disparities and promoting the growth of a sustainable health workforce.

## Building place based education and research

With a focus on social accountability, Fuller, Beattie, McGrail et al.' scoping literature review identified 138 articles on place-based preregistration HPE, with 50 unique HPE programs (predominantly medicine, nursing and midwifery) from 12 countries. Place-based programs were characterized by three common features closely aligned with social accountability: widening access to HPE; comprehensive program design; and a community-engaged approach.

Turning to research, Schmidt et al. in Australia undertook a focused Systematic Review in the form of a realist synthesis that demonstrated rural workplace-based research training is effective, but not sufficient to build and maintain rural health research capacity. Addressing both structural and individual factors is needed to build rural health research capacity and generate real-world health research to drive meaningful improvements in rural health. These findings are echoed by Welton et al. in Canada whose Perspective presents the case for rural health research to be undertaken in rural communities, by rural communities, for rural communities.

## Immersive community engaged education including Longitudinal Integrated Clerkships

The majority of articles in this Research Topic highlight the importance of prolonged place-based undergraduate HPE and postgraduate training that occurs predominantly in remote and rural community settings.

Rural family practice based Longitudinal Integrated Clerkships (LICs) are well-established examples of immersion whereby students are living in a rural community and learning their core clinical medicine from the family practice, community perspective (10, 11). In Canada, Kelly et al. explored the students' experience of LICs. They found that, beyond continuity of relationships with preceptors and patients, factors such as personal relationships, community connections, learning in a resource-strained environment, geographical isolation, and other socio-political dynamics, impacted the LIC learner experiences of continuity and community integration. The Scottish Graduate Entry Medicine (ScotGEM) program features a LIC as students' principal clinical year in rural general practice (Graham et al.). There are signs that ScotGEM graduates are more likely to choose rural careers and primary care than graduates from other Scottish medical programs.

The length and the depth of immersion were shown to contribute to impact. In Australia, Harvey, Ali et al. found that, when compared to 1 year, graduates of 3 years of rural immersion were significantly more likely to practice in remote, rural or regional areas. The South African experience of rural homestays highlighted the value of deeper immersion whereby students live with local families during their 7-week rural placement. Gaede found that, in addition to placed-based educational value, the homestays showed a strong potential for humanizing the professional development of students, underpinned by active authentic relationships between students and community members.

## Scalable distributed postgraduate training

In the Philippines, Espina et al.' Community Case Study presents the design and implementation of the Sorsogon Province-wide Practice-Based Family and Community Residency Training Program a distributed, *in-situ* model co-developed by the Sorsogon Provincial Government and the Philippine Academy of Family Physicians. Over 4 years, the program has matured to achieve full accreditation status and has successfully prepared its trainees to lead primary care delivery in resource-constrained, community-based settings. This case highlights the feasibility of scaling practice-based residency training models in LMICs through strong local governance, policy support, and community-responsive curriculum design.

The Australian Remote Vocational Training Scheme (RVTS) provides a scalable approach to rural general practice training that utilizes distance education and remote supervision. Giddings et al. report that RVTS enables trainees to stay in their rural, remote and Indigenous communities while working toward specialist certification as a general practitioner. Also in Australia, Seidu et al. explored Additional Skills Training (AST) for rural generalist practice and found that AST is a highly valued by GPs involved in the program who were intrinsically motivated to participate. However, to ensure its sustainability, wider recognition of the value, better visibility, and better alignment with community needs are required.

## Rural educational innovations

Curriculum innovations are a major feature of ScotGEM (Graham et al.), specifically: the Generalist Clinical Mentor (GCM) role; the year-long GP based LIC; and the Agents of Change (AoC) course. The GCM role involves employing clinical generalists in a multi-dimensional clinical-academic role providing mentorship, teaching and clinical services within university and affiliated clinical teaching practice settings. ScotGEM's underlying social accountability ethos led to the inclusion of AoC to help students develop the skills, knowledge and mindsets to drive positive change in diverse healthcare systems throughout their medical careers.

In Canada, Perez et al. present peer-led learning as an effective innovation whereby a rural background student organized a rural day-long educational excursion to their own community. They found that: informal teaching facilitated learning; trust in their peer enabled students to receive information more favorably; and students gained a better understanding of rural life and medical practice. Another Canadian innovation is Living Library story telling that was developed to provide students with better understanding of rural life and practice through narratives. Perez Malhi et al. found that the stories allowed students: to walk in a rural professional's shoes, enabling them to see “rural” in a new light; and to self-reflect and gain a sense of personal growth.

## Rural career pathways

Medical schools in Croatia largely continue the 20th century model of medical education with a lack of curricula content on rural medicine or specialized training for rural practice. Mrduljaš-Đujić et al. explored the perceptions of first and final year medical students and found that final year students felt insufficiently prepared for rural practice, although rural background students showed more interest in practicing outside the main cities.

The Alabama Rural Health Leaders Pipeline (ARHLP) developed over 25 years, specifically to support rural students, including from the Black Belt, through an education pathway into and through medical school toward a career in rural primary care. Wheat reports that, compared to peers in traditional medical education, ARHLP graduates more frequently chose family medicine specialty and rural Alabama practice with no difference in academic performance, although few graduates are practicing in the Black Belt.

In Australia, two universities cooperated to introduce an end-to-end rural medical pathway with a place-based curriculum and distributed medical education (DME) model. Harvey, Van Schaik et al. report on the practical aspects of developing and delivering this pathway with the expectation that the program will foster long-term professional and personal ties to rural communities and prove to be a scalable and evidence-based model for addressing rural medical workforce shortages.

Another Australian rural medical pathway is reported by Fuller, Beattie, Versace et al. in the form of the place-based Rural Training Stream (RTS) including a rural LIC that was established (2022). The RTS involves recruitment of students from the medical school's designated rural footprint and supporting these students through local DME to graduation. Baseline findings suggest that RTS graduates may be more likely to stay in the rural footprint for up to 2 years, however subsequent attrition suggests the need to continue the pathway into and beyond postgraduate training.

## Conclusion

This Research Topic of articles adds value to the published literature specifically providing further evidence of the advantages of place-based, immersive community engaged education, cradle-to-grave facilitated rural career pathways, distributed postgraduate training for rural practice, and community engagement in rural health research, HPE and health service delivery (12). Drawn from eight countries in five continents, the articles confirm that, despite obvious differences between countries, remote rural communities across international borders face similar challenges and opportunities to improve healthcare and HPE. Despite this global breadth, contributions from LMICs were limited, underscoring barriers such as publication fees and English-language dominance. Addressing this gap is essential if the global discourse on distributed HPE is to be truly inclusive.

Although many distributed HPE programs are well-established, there still is a need for more data on long-term outcomes to establish what common features that are most likely to lead to long term rural health workforce stability. In addition, there is a need for greater training in all specialties in rural areas to sustain a fit-for-purpose workforce in larger rural and regional centers.

People living in remote rural communities and their healthcare providers are experts on themselves, so it is important to respect and value this expertise through co-creation, co-development, co-delivery and co-evaluation of education, training, research and health service delivery. Guided by social accountability and community engagement, this “start local” approach employs a strengths-based framework to enhance rural community capacity building, toward ensuring people in remote rural communities have access to high-quality healthcare delivered by locally trained health professionals (13).
